# Immunogenicity and protective efficacy of nanoparticle formulations of L-SseB against *Salmonella* infection

**DOI:** 10.3389/fimmu.2023.1208848

**Published:** 2023-06-30

**Authors:** Sayan Das, Debaki R. Howlader, Ti Lu, Sean K. Whittier, Gang Hu, Simran Sharma, Zackary K. Dietz, Siva S. K. Ratnakaram, David J. Varisco, Robert K. Ernst, William D. Picking, Wendy L. Picking

**Affiliations:** ^1^ Department of Pharmaceutical Chemistry, University of Kansas, Lawrence, KS, United States; ^2^ Department of Veterinary Pathobiology and Bond Life Science Center, University of Missouri, Columbia, MO, United States; ^3^ Department of Microbial Pathogenesis, University of Maryland, Baltimore, MD, United States

**Keywords:** Salmonella, T3SS, vaccine, formulation, typhoid, IL-17

## Abstract

*Salmonella enterica*, a Gram-negative pathogen, has over 2500 serovars that infect a wide range of hosts. In humans, *S. enterica* causes typhoid or gastroenteritis and is a major public health concern. In this study, SseB (the tip protein of the *Salmonella* pathogenicity island 2 type III secretion system) was fused with the LTA1 subunit of labile-toxin from enterotoxigenic *E. coli* to make the self-adjuvanting antigen L-SseB. Two unique nanoparticle formulations were developed to allow multimeric presentation of L-SseB. Mice were vaccinated with these formulations and protective efficacy determined *via* challenging the mice with *S. enterica* serovars. The polysaccharide (chitosan) formulation was found to elicit better protection when compared to the squalene nanoemulsion. When the polysaccharide formulation was used to vaccinate rabbits, protection from *S. enterica* challenge was elicited. In summary, L-SseB in a particulate polysaccharide formulation appears to be an attractive candidate vaccine capable of broad protection against *S. enterica.*

## Introduction

*Salmonella enterica* is a Gram-negative pathogen that is responsible for causing diseases having diverse clinical manifestations in humans, pets, livestock, and poultry. The species has over 2500 serovars that can infect a wide variety of hosts. According to the World Health Organization, non-typhoidal *S. enterica* is responsible for infecting one in ten people at a loss of 33 million healthy life years annually. In the United States, it is responsible for numerous outbreaks of gastroenteritis due to consumption of contaminated food (1) with 30-50% of bacterial foodborne infections attributable to *S. enterica*. Fortunately, the disease in self-limiting in healthy individuals and is resolved quickly (2); however, in immune-compromised individuals the infection can often be life-threatening (3). In the U.S., treatment is complicated by the emergence of multi-drug resistant strains with a 40% increase in resistant strain infections in 2015-2016 compared to 2004-2008 (4). Typhoid fever caused by *S. enterica* serovars Typhi and Paratyphi are responsible for a disease characterized by fever, organomegaly and, in severe cases, intestinal hemorrhaging or perforation, if left untreated. Globally, the disease was responsible for 131,200 deaths in 2017 (3).

Cases of the more serious invasive non-typhoidal *Salmonella* (iNTS) have been on the rise globally. There were 535,000 iNTS cases and ~77,500 deaths reported in 2017 with the highest incidence in Sub-Saharan Africa (3). The disease has a mortality rate of ~20% which may increase substantially in patients infected with HIV (5, 6). Treatment in the Sub-Saharan region is complicated by the fact that disease symptoms at onset are often similar to commonly occurring comorbidities (e.g. malaria and pneumonia) (7, 8). Although iNTS can be treated successfully with antimicrobials, emerging multidrug-resistant (MDR) strains are becoming a major concern (9).

Vaccination is perhaps the most effective way of treating the disease and preventing the spread of MDR strains. Currently there are two licensed vaccines available and recommended for use against typhoid fever: a live attenuated vaccine (Ty21a) and a capsular polysaccharide-based vaccine (Vi) (10). Several *S.* Typhimurium glycoconjugate vaccines are also being developed with an aim to generate protective antibody responses against the core and O-polysaccharide of lipopolysaccharide (LPS) present in the bacterial outer membrane. Conjugating these carbohydrate components to a protein carrier converts the resulting T cell-independent response into a T cell-dependent one (11–13). It should be noted that much of the immunity generated by conjugate vaccines has been attributed to the resulting antibody responses; however, the role of antibodies in preventing infections outside a narrow range of *Salmonella* serovars (e.g. typhoidal and nontyphoidal serovars) is questionable (14). Furthermore, these vaccines have been shown to be moderately protective (32% for Ty21a and 52% for Vi capsular polysaccharide vaccine) in humans (15, 16) Although these antibodies reduces the severity of the disease, they are unable to protect against the actual infection (17). Considering these facts, a protein subunit vaccine that can potentially recognize a broad range of *Salmonella* serovars would represent an important public health achievement.

In this study, SseB, which is a highly conserved protein localized at the tip of SPI-2 type III secretion apparatus (T3SA) needle, is used as the antigen. Mutations within the individual proteins of the T3SA are not tolerated well and result in non-functional assemblies that compromise pathogen virulence. Therefore, vaccine escape is unlikely for these vaccine targets. SseB was identified as a common target by McSorley et al. (18), however, we have not been able to elicit strong protection in our animal models using SseB alone when administered at a mucosal site using the mucosal adjuvant dmLT (double-mutant labile toxin from enterotoxigenic *E. coli*). Therefore, based on parallel work from our laboratory, SseB was genetically fused with the A1 subunit of dmLT to generate self-adjuvating L-SseB. L-SseB circumvents the use of dmLT as an adjuvant and the observation that it can lead to cause Bell’s palsy (19). We then elected to formulate L-SseB into a nanoparticle (NP) to enhance its presentation to the innate immune response and to include the TLR-4 agonist BECC438b (20) (Bacterial Enzyme Combinatorial Chemistry candidate 438 extracted from an engineered *Yersinia* background) which is a biosimilar of the adjuvant monophosphoryl lipid A (MPL).

The efficacy of L-SseB in the two formulations, MedImmune emulsion (ME) (a squalene-based oil-in water emulsion) or Chi-C48/80 (a particulate polysaccharide NP) with and without BECC438b was then determined. We then tested the dose response of this vaccine and found that it elicited robust cell-mediated immunity, which is essential for prevention and clearance of *Salmonella* (14). This formulation was able to protect against *S.* Typhimurium and *S.* Typhi in a mouse model indicating effectiveness against typhoidal and non-typhoidal (including iNTS) *Salmonella* infections. Protection was then validated in a rabbit diarrheal model of *S.* Typhimurium infection and found to be effective. In summary, a chitosan-based NP vaccine containing an LTA1 mucosal adjuvant and a TLR-4 agonist shows potential to be a successful broadly protective vaccine against *Salmonella* infections.

## Materials and methods

### Materials

pET15b plasmid, ligation mix, and competent Tuner DE3 *E. coli* were from Millipore-Sigma (St. Louis, MO). Chromatography columns were from GE Healthcare (Piscataway, NJ). All other reagents were from Sigma or Fisher Scientific and were chemical grade or higher. dmLT was a gift from J. Clements and E. Norton (Tulane School of Medicine, New Orleans, LA) and *Salmonella enterica* serovar Typhimurium SL1344 was a gift from Jorge Galan (Yale University, New Haven, CT). *S.* Typhi (strain 700931) was purchased from ATCC (Manassas, VA). Squalene was purchased from Echelon Biosciences (Salt Lake City, UT).

## Methods

### Protein production

The coding sequence for SseB was ligated into pET15b (Millipore-Sigma, St. Louis, MO). The coding sequence for LTA1 was inserted 5’ to the SseB as described previously (21). The plasmid was used to transform *E. coli* Tuner (DE3). Bacteria were grown in LB broth with shaking at 200 rpm until the A_600_ reached 0.8. Protein expression was induced by adding IPTG to 1 mM and the culture incubated for an additional 3 h. The bacteria were collected by centrifugation, resuspended in 1X His-Tag Binding Buffer (20 mM Tris-HCl pH 7.9, 500 mM NaCl, 10 mM imidazole), and passed through a LM10 microfluidizer (Microfluidics, Westwood, MA) three times at 18,000 PSI. Because the L-SseB sequestered to inclusion bodies, the lysed bacteria were clarified at 10,000 x g and the pellet containing the inclusion bodies was retained. The pellet was resuspended in 8M urea in 1X His-Tag Binding Buffer. The solution was clarified at 15,000 x g and the supernatant saved. The L-SseB was then purified as previously described for IpaD except 8M urea was present during the entire process (22, 23) The eluted L-SseB was refolded by stepwise dialysis into 10 mM phosphate pH 7.2, 150 mM NaCl (PBS) with the urea reduced by 2M at each buffer exchange. The L-SseB was stored at -80°C (**Supplementary Figure 1A**). LPS levels were determined using a NexGen PTS with EndoSafe cartridges (Charles River Laboratories, Wilmington, MA). All proteins had LPS levels <5 Endotoxin units/mg protein.

### ADP-ribosylation assay

LTA1 from labile toxin of enterotoxigenic *E. coli* possesses ADP-ribosylase (ADPr) activity, which is required for adjuvanticity (19). Because the L-SseB was isolated from inclusion bodies after urea denaturation and refolding, it was important to assess the activity of the LTA1 moiety (**Supplementary Figure 1B**). The activity assay was carried out in a 15 µl reaction mix containing 50 mM Tris-HCl pH 7.5, 1 mM EDTA, 1 mM DTT, 500 ng ARF4 substrate, 8.3 µM biotin-labeled NAD^+^ (BPS Biosciences, San Diego, CA) and 2500 ng of LTA1 fusion protein. After a 1 h incubation at 37^°^C, the reaction was stopped by adding 5 µl of 3X SDS-PAGE sample buffer and boiled for 5 min. Proteins were separated by 15% SDS-PAGE, subjected to western blot analysis with the biotin moiety on the ribosylated proteins visualized with IR dye 800 CW Streptavidin (Li-Cor, Lincoln, NE).

### Far-UV circular dichroism (CD) spectroscopy

CD Spectra in the far UV region were obtained using a Chirascan-plus CD Spectrophotometer (Applied Photophysics, UK) equipped with a Peltier controlled six cell holder. L-SseB (0.1 mg/mL) was loaded into a 1-mm quartz cuvette and spectra were obtained in the far UV region (190 to 260 nm) in 1nm increments with 1 sec per data point integration time. To evaluate thermal melting, a temperature ramp was imposed from 10 to 90°C (acquisition at every 2.5°C) with an equilibration of 2 min at each temperature. After acquisition at 90°C, the L-SseB protein samples were cooled to 10°C and rescanned (**Supplementary Figure 2**).

### Determining the hydrodynamic diameter using dynamic light scattering

The hydrodynamic diameter (Dh) of the L-SseB and formulations were determined by dynamic light scattering (DLS) using a Zetasizer Ultra (Malvern Instruments) (24). Formulations were diluted in 1:10 with water in triplicate and measured in disposable polystyrene cuvettes. The parameters used were as follows: material RI = 1.59, dispersant RI (water) = 1.33, T = 25°C, viscosity (water) = 0.887 cP, measurement angle = 173° backscatter with automatic attenuation. The Z-average values of the Dh for these samples were calculated *via* cumulant analysis.

### Preparation of vaccine formulations

The two vaccine preparations were prepared as described previously (24). Briefly, to make ME (MedImmune Emulsion), squalene (8% by weight) and polysorbate 80 (2% by weight) were mixed to achieve a homogenous oil phase and 40 mM Histidine (pH 6) with 20% sucrose was added to the oil phase to generate 4XME (25). To make the L-SseB with ME, the protein was added to the emulsion to a final concentration of 0.67 mg/ml, vortexed and allowed to incubate overnight at 4°C. The association of L-SseB was assessed using dynamic light scattering (DLS) as described elsewhere (24). BECC438b was made and resuspended as previously described (20). To make BECC438b+ME, BECC438b was mixed by vortexing for 2 min with the ME and incubated overnight at 4°C. The next day, L-SseB was added at a volumetric ratio of 1:1 to achieve the desired final antigen concentration.

Chitosan (Chi) nanoparticles were made by washing the chitosan and then loading it with C48/80 as described (24). The L-SseB in PBS was exchanged into 20 mM histidine buffer pH 6. To make L-SseB-Chi-C48/80, L-SseB was then added in a 1:4 w/v ratio, vortexed and incubated for 2 h at 4°C. Unbound L-SseB was removed by washing the nanoparticles once. Binding efficiency was determined to be 75% based upon the A_280_ remaining in the supernatant after removal of the particulate chitosan fraction. Where BECC438b was included, the nanoparticles with L-SseB were mixed with BECC438b by vortexing and incubating overnight at 4°C prior to addition the protein.

### Mice and immunizations

The animal protocols were reviewed and approved by the University of Kansas Institutional Animal Care and Use Committee Practices (protocol AUS 222-01). Six- to eight-week-old Balb/c mice (Charles River Laboratories, Wilmington, MA) were used for all experiments. For immunizations, mice were anesthetized with isoflurane and vaccine formulations administered intranasally (IN) as previously described (23). Immunizations were on days 0, 14, and 28 for this study. As a positive control, a group of mice was orally administered a live attenuated *Salmonella* strain (*aro*) on days 0 and 14 (26). Blood was collected prior to vaccination and on days 42 and 56. No adverse reactions were observed for any of the antigens or vaccine formulations tested here.

### *Salmonella* infection

For *S.* Typhimurium infection, mice were starved for 4 h and infected with SL 1344 by oral gavage after neutralization of gastric acids with 0.1 ml 5% NaHCO_3_ 15 min prior to bacterial challenge. For the survival assay, mice were challenged with logarithmically grown cultures of 1×10^6^ CFU/mouse of *S*. Typhimurium and monitored for clinical signs of infection (fever, reduced locomotion, piloerection), morbidity and mortality over 36 days. Morbid mice were humanely euthanized.

An iron overload mouse model was used for oral infection with *S*. Typhi as described previously (27). Mice were injected intraperitoneally (IP) with deferoxamine (0.5 mg/gm body weight) and ferric chloride (0.32 mg/gm body weight). Mice were starved for 4 h, gastric acids neutralized with 0.1ml 5% NaHCO_3_ and the mice challenged *via* intragastric route by *S.* Typhi (1×10^6^ CFU/mouse).

To determine visceral bacterial load, mice were infected with sub-lethal doses of *S*. Typhimurium (3x10^5^ CFU/mouse) or *S*. Typhi (1x10^4^ CFU/mouse). Liver and spleen were harvested at desired time points. Organs were homogenized and lysed with 0.1% Triton X-100. The bacterial load was enumerated via serial dilution of the lysates and spread on Luria agar plates containing streptomycin (50 μg/ml).

### Tissue resident memory T cells (TRMs)

Each mouse was intravenously injected with a Treg protector (50 μg/mouse; Biolegend) and euthanized 30 min post injection (28). The liver was extracted and processed through a 70-micron cell strainer into a 50 ml conical tube. The tube was filled with 40 ml RPMI 1640 and the solution clarified at 2000 x *g* for 10 mins without braking. The supernatant was discarded, the pellet re-suspended in 15 ml of Percoll and centrifuged at 2000 x *g* for 10 mins without braking. The top layer of cells was discarded while the cells at the interface were collected and washed with PBS containing 2% FBS. Erythrocytes were lysed with lysis buffer and the cells were resuspended in PBS containing 2% FBS for counting. Cell concentration was adjusted to 1x10^6^/tube and stained with fluorochrome tagged antibodies against CD4, CD69 and P2X7. The cells were incubated on ice for 30 min and washed with PBS containing 2% FBS. The cells were then analyzed via flow-cytometry (BD FACS Aria III Fusion).

### Cytokine determinations

Cells isolated from liver and spleen were incubated with 10 µg/ml SseB or PBS for 48 h at 37°C. Supernatants were collected and analyzed for cytokines: IFN-γ, IL-10, IL-12p70, IL-17A, IL-1β, IL-2, IL-4, IL-5, IL-6, and TNF-α with concentrations being determined using an MSD plate reader with associated analytical software (Meso Scale Discovery, Rockville, MD). While multiple cytokines were measured, only the ones that were differentially modulated upon stimulation are reported here.

### Rabbit studies

Anesthetized New Zealand White (NZW) rabbits (~6 weeks old, 6/group) were immunized IN (days 0, 14 and 28) with PBS, 75 µg or 200 µg L-SseB-Chi-C48/80+BECC438b. On day 56 post first immunization, rabbits were orally challenged with 1x10^5^ CFU of *Salmonella* Typhimurium/rabbit (29) following neutralization of gastric acidity with 15 ml of 5% sodium bicarbonate. This dose was chosen as it was below the dose threshold that caused diarrhea in the rabbits. They were then monitored for 15 days, and the body weights were recorded. Rabbits were euthanized if diarrhea was detected as the goal was to prevent diarrhea.

### Isolation of Rabbit PBMC and cytokines analysis

On day 42, blood was collected from the center auricular artery of the ear. Blood (1 ml) was diluted to 7 ml using PBS and layered over 3 ml histopaque 1077 (Sigma) in a 15 ml conical tube. The tube was centrifuged at 400 x *g* for 35 mins (no braking) and the cells from the interface were collected. After washing with PBS containing 2% FBS the cells were resuspended in complete RPMI 1640. The cells were enumerated and seeded into 96 well tissue culture plates (1x10^5^ cells/well) and stimulated with SseB (2 μg/well) or left unstimulated. The plates were incubated at 37°C, 5% CO_2_ for 24 h. The supernatants were collected and analyzed for the presence of IFN-γ and IL-17A by ELISA (Invitrogen) according to the manufacturer’s instructions.

### ELISA

Antibodies specific for SseB were determined by ELISA as described previously with minor modifications (21, 23). Briefly, 96-well plates coated with SseB (1 µg/mL in PBS) were blocked overnight with 10% nonfat dry milk in PBS. Each well was incubated with serum samples for 1 h at 37°C. After washing the plates with PBS-Tween 20 (0.05%), secondary antibody (1:3000 KPL, Gaithersburg, MD) was added and incubated for 1 h at 37°C. Level of IgG (H+L) was determined with horseradish peroxidase-conjugated secondary antibodies (human serum adsorbed) generated in goats (Southern Biotech, Birmingham, AL). 3,3′,5,5′-Tetramethylbenzidine (TMB) substrate was added, and reaction was stopped with H_3_PO_4_. Endpoint titers were calculated and represented as ELISA units per ml (EU ml^-1^).

### Statistical analysis

GraphPad Prism 8.1.2 was used to perform all statistical comparisons. Differences among treatment groups were analyzed as mentioned in figure legends. A *p* value of less than 0.05 was considered significant for all comparisons. * P< 0.05; ** P<0.01; *** P< 0.001.

## Results

### Evaluation of protective efficacy of L-SseB and formulations

We have previously reported on the protective efficacy elicited in mice vaccinated with a fusion protein consisting of the needle tip and first translocator fusion proteins from the SPI-1 (SipD and SipB) and SPI-2 (SseB and SseC) T3SS (30). While successful in protecting mice from death caused by *Salmonella* infection, this protein cocktail was onerous to produce, and these particular proteins were only marginally stable and would be difficult to produce on a large scale. Based on reports from other groups (31) and the success of our T3SS-based vaccine platform, we decided to fuse LTA1 to SseB to give L-SseB (21, 32) to generate a stable self-adjuvanting subunit vaccine containing only a single T3SS component. Because L-SseB sequestered to inclusion bodies during expression in *E. coli*, it was purified under denaturing conditions, so to ensure our renaturation methods were successful, we tested and found that the fusion had retained LTA1 moiety’s ADPr activity (**Supplementary Figure 1**). Likewise, when analyzed by circular dichroism (CD) spectroscopy, the fusion protein was shown to possess an appropriate secondary structure content with mixed α-helical and β-strand makeup and was thermally stable to greater than 60°C (**Supplementary Figure 2**). Once we were confident that L-SseB could be stably produced, we formulated into two unique candidate vaccines. One formulation was a squalene-based oil-in-water emulsion (ME) and the second was a polysaccharide-based (chitosan) nanoparticle. Each of these formulations allowed for the presentation of the antigen in a multimeric form (21). Additionally, we also tested the effect of an additional adjuvant called BECC438b, a lipid A analogue that is a biosimilar of monophosphoryl lipid A and a TLR-4 agonist, to determine how it might contribute to any observed protective responses.

### L-SseB interacts with ME

The first formulation examined was an oil-in water emulsion referred to as ME (MedImmune Emulsion) (24). ME is typically in the 100-200 nm size range and thus can readily be taken up directly by dendritic cells (33, 34). We determined that L-SseB associated with ME+BECC438b by measuring the particle size distribution of ME+BECC438b before and after mixing with L-SseB. L-SseB had a peak mean by intensity (nm) of 393.2 ± 61.5 with a polydispersity index of 0.627 ± 0.079. The relatively larger size of L-SseB is believed to be a reflection of the SseB component which is known to form an oligomer at the SPI-2 T3SA needle tip structure (35). Conversely, ME+BECC438b had a unimodal size distribution of 232 ± 8.7 with a low polydispersity index of 0.268 ± 0.015. Mixing L-SseB with ME+BECC438b slightly increased the nanoemulsion particle size to 244 nm without an increase in polydispersity (0.195 ± 0.02). This suggests that individual L-SseB molecules associate with the ME surface rather than complete L-SseB oligomers. The addition of BECC438b to the ME particles did not noticeably change the size of the nanoemulsion particle (data not shown).

### L-SseB interacts with Chi-C48/80 (Chi)

As a second unique particulate formulation, L-SseB was bound to chitosan. Chitosan nanoparticles are a potentially attractive delivery particle due to their positive charge which allows them to interact with negatively charged polysaccharides such as mucin, which is the major component of mucus layer on the surface of nasal epithelial cells. In prior work, DLS measurements were used to confirm the interaction of our preparation of Chi with mucin (24). We thus used DLS to determine the influence that L-SseB association has on the size of the Chi particles. Chi alone has a Dh of 1573 ± 366 nm with a high polydispersity value (0.785), however, the addition of BECC438b reduced the apparent size to 682 ± 246 nm though the polydispersity was still rather high (0.938). The subsequent addition of L-SseB cause a further reduction in size to 293 ± 45 nm and improved the polydispersity value to 0.378. The addition of BECC438b and L-SseB appeared to cause a reduction in Chi aggregation and resulted in a particle that would be amenable to uptake by antigen presenting cells.

### Evaluation of protective efficacy of L-SseB and formulations

**Figure 1:**
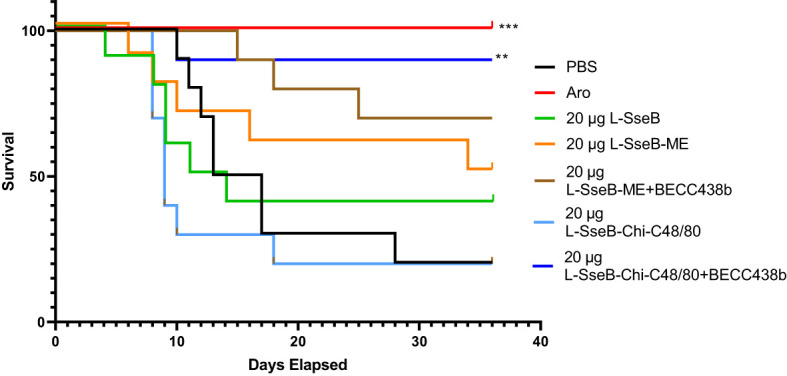
Evaluation of protective efficacy of L-SseB and formulations. Balb/C mice (n=10/group) were vaccinated orally with a *S.* Typhimurium live, attenuated (*aro*) strain (positive vaccine control) on days 0, 14 or intranasally (IN) for all other L-SseB-containing vaccine formulations on days 0, 14 and 28. On day 56 mice were challenged orally with *S.* Typhimurium (1 x 10^6^ CFU/mouse). Significance was calculated by comparing survival of individual vaccinated groups with the PBS vaccinated (negative vaccine control) group using Log-rank (Mantel-Cox) test. **p<0.01, ***p<0.001.

Initially, we vaccinated mice with L-SseB alone or as part of the ME or Chi-C48/80 formulations with or without BECC438b to determine the resulting antibody responses. The vaccine positive control for these experiments was a *S.* Typhimurium live attenuated *aro* strain. Strong IgG responses were detected in all the groups vaccinated with L-SseB but not in either the negative control (PBS) or positive vaccine control *aro* groups (**Supplementary Figures 3-5**). The protective efficacy of the vaccine formulations was assessed using a *S.* Typhimurium typhoid-like mouse model (**Figure 1**). These mice were vaccinated IN three times with 20 µg L-SseB alone, or ME ± BECC438b or Chi-C48/80 ± BECC438b. The positive control was the live attenuated *aro* strain (26). On day 56, the mice were orally challenged with 1x10^6^ *S.* Typhimurium and monitored for 36 days (**Figure 1**). As expected, the *aro* strain protected all mice from the *S.* Typhimurium challenge. The L-SseB alone was only 25% protective when compared to the PBS negative control. When L-SseB was added to ME, the protection increased slightly to 38%, but this was further increased to 63% with the addition of BECC438b. The protective efficacy of the L-SseB-Chi-C48/80 was the same as the negative control, however, the addition of BECC438b resulted in a significant increase in the protective efficacy to ~88%.

### Cytokine production in response to stimulation with SseB

**Figure 2:**
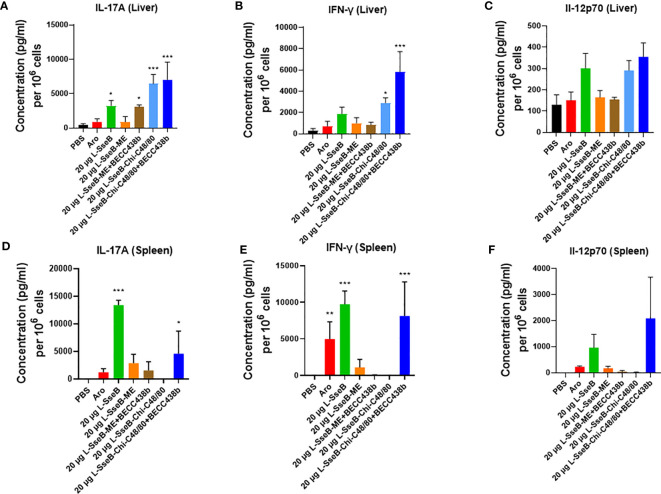
Cytokine production by isolated cells after stimulation with SseB. Single cell suspensions of livers **(A–C)** and spleens **(D–F)** from mice vaccinated as in **Figure 1** were stimulated with SseB as described in Methods. The supernatants were analyzed for cytokine production by MesoScale Discovery (MSD) analysis as per the manufacturer’s specifications. Significance was calculated by comparing cytokine secretion by cells isolated from PBS vaccinated mice to all other vaccinated groups using one-way ANOVA (Dunnett’s multiple comparison test). *p<0.05, **p<0.01, ***p<0.001.

The SseB specific cytokine response was evaluated in cells isolated from the livers and spleens of mice vaccinated as above (**Figure 1**). Single cell suspensions were stimulated with SseB or left unstimulated with the supernatants then analyzed for secreted cytokines after incubating for 48 h (**Figure 2**). SseB induced significant levels of IL-17A secretion in mouse livers of the L-SseB alone, with ME+BECC438b or with Chi-C48/80 ± BECC438b (**Figure 2A**). In contrast, only the L-SseB alone or with Chi-C48/80+BECC438b groups displayed significant levels of IL-17A secreted from the spleen cells stimulated with SseB (**Figure 2D**). With respect to IFN-γ, groups immunized with L-SseB-Chi-C48/80 ± BECC438b had significant levels of the cytokine secreted from the liver (**Figure 2B**). In contrast, groups immunized with the *aro* strain, L-SseB alone or L-SseB-Chi-C48/80+BECC438b had significant levels of IFN-γ secreted from splenocytes (**Figure 2E**). Interestingly, the only group that showed a significant level of protection also demonstrated a significant increase in secretion of IL-17A and IFN-γ in liver and spleen cells. All vaccinated groups that were not protected from *S.* Typhimurium challenge were lacking significant secretion of either IL-17A or IFN-γ (or both) in the cells from the liver or spleen. While there was increased secretion of IL-12p70 in both liver and spleen in some groups, the differences were not statistically significant (**Figures 2C, F**).

### Induction of tissue resident memory T cells in response to vaccination

**Figure 3:**
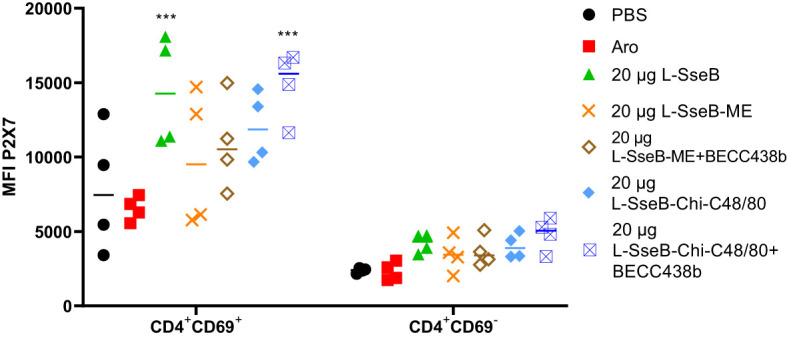
Induction of tissue resident memory (TRM) T cells in response to vaccination. Balb/C mice (n=4/group) were vaccinated as already described. On day 56, mice were euthanized, and single cell suspensions were prepared from livers. Isolated cells were co-stained with fluorochrome-conjugated CD4, CD69 and P2X7 antibodies and analyzed by flow-cytometry. Mean Fluorescence Intensity (MFI) of P2X7 stained cells was plotted. Significance was calculated by comparing the MFI for P2X7 in the PBS vaccinated group to all other groups by using one-way ANOVA (Dunnett’s multiple comparison test). ***p<0.001.

The induction of tissue resident memory T cells (TRMs) generated by the mice vaccinated as described above was evaluated next (**Figure 3**). Single cell suspension from livers of immunized mice were stained with fluorochrome conjugated antibodies against CD4 (a T helper cell marker), CD69 (an early activation marker) and P2X7 (a receptor promoting inflammatory responses). The mean fluorescence intensity (MFI) of P2X7 among the groups was compared (**Figure 3**). Groups immunized with L-SseB and L-SseB-Chi C48/80+BECC438b showed significant increases in P2X7 MFI (for the CD4^+^CD69^+^ cell population), as compared to the PBS immunized group. Vaccination with both L-SseB and L-SseB-Chi C48/80+BECC438b induced TRMs; however, the former was not able to generate acceptable protection supporting the proposal that TRMs are indispensable but not the only factor contributing to protection.

### Cytokine production and TRMs in response to stimulation with SseB in mice immunized with increasing L-SseB concentrations

**Figure 4:**
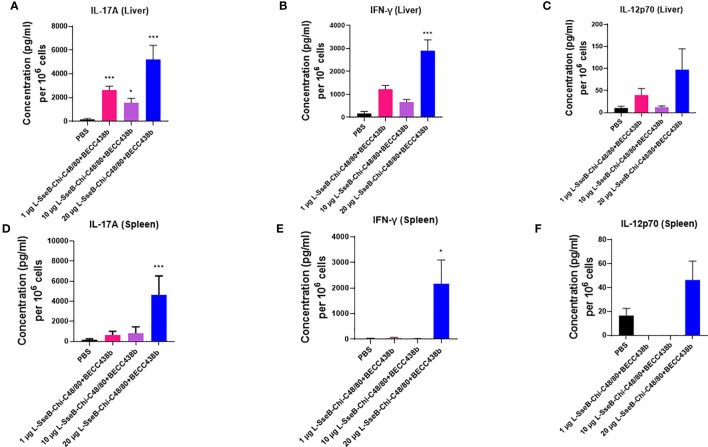
Cytokine production by isolated cells after stimulation with SseB for mice immunized with increasing L-SseB concentrations. Balb/C mice (n=5/group) were IN vaccinated with PBS or 1, 10 or 20 µg L-SseB in Chi-C48/80-BECC438b on days 0,14 and 28. On day 56, mice were euthanized, and single cell suspensions were prepared. Single cell suspensions of livers **(A–C)** and spleens **(D–F)** were stimulated with SseB, and the supernatants analyzed for cytokine production using MSD analysis as per manufacturer’s specifications. Significance was calculated by comparing cytokine secretion by cells isolated from PBS vaccinated mice to all vaccinated groups by using one-way ANOVA (Dunnett’s multiple comparison test). *p<0.05, ***p<0.001.

**Figure 5:**
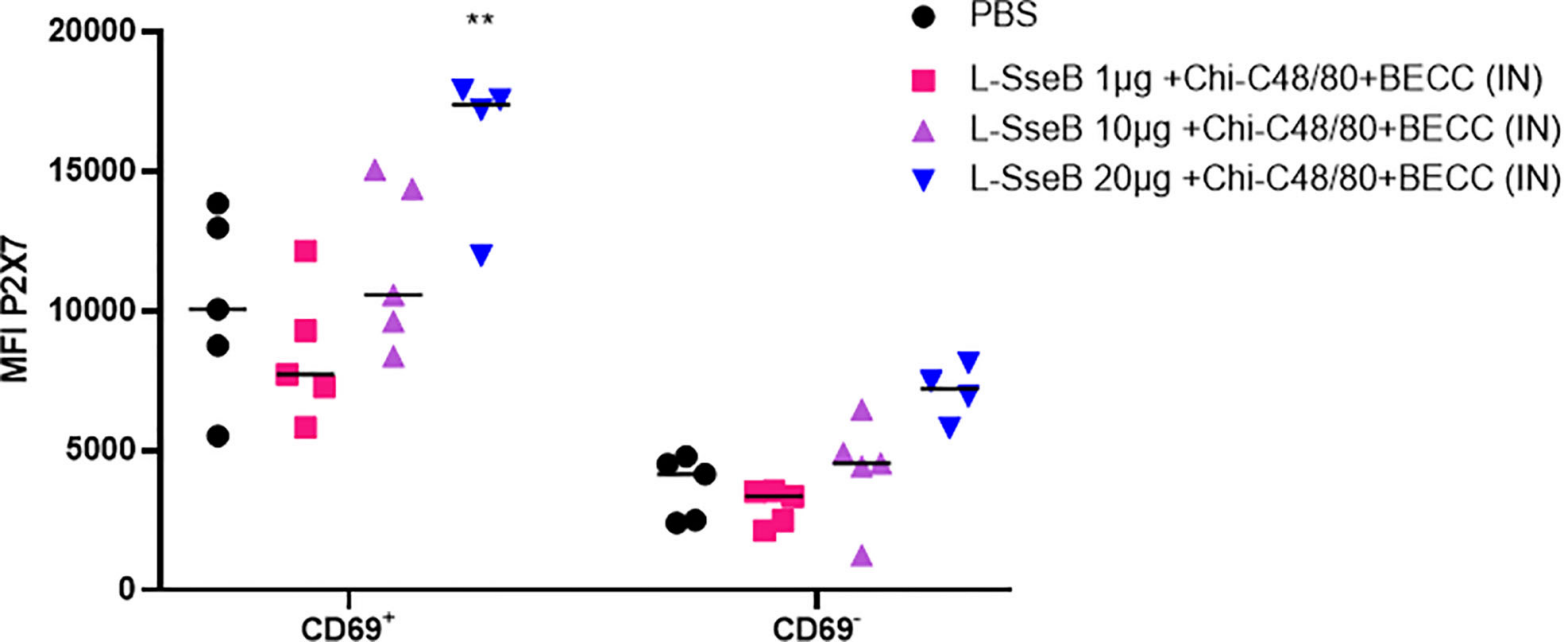
Induction of tissue resident memory T cells in response to dose escalation of L-SseB. Isolated cells from the groups described in **Figure 4** were co-stained with fluorochrome conjugated CD4, CD69 and P2X7 antibodies. Mean Fluorescence Intensity was analyzed by flow-cytometry and the MFI of P2X7 stained cells was plotted. Significance was calculated by comparing the MFI of P2X7 in PBS vaccinated group to all other groups by using one-way ANOVA (Dunnett’s multiple comparison test). **p<0.01.

Having determined that L-SseB-Chi-C48/80+BECC438b was the lead protective formulation of those tested here, we performed a dose escalation in mice using 1, 10, and 20 µg of L-SseB with Chi-C48/80+BECC438b. After three vaccinations, mice were rested for four weeks and then the livers and spleens harvested. Single cell suspensions were produced from these organs, and these were stimulated with SseB to determine the secretion levels of IL-17A, IFN-γ, and IL-12p70 (**Figure 4**). As shown above, the 20 µg L-SseB-Chi-C48/80+BECC438b group showed a significant increase in the levels of secreted IL-17A and IFN-γ from both liver and spleen cells. High levels of secreted IL-12p70 were also seen in this group of mice (**Figure 4**). The induction of TRMs was also examined and only the group immunized with 20 µg L-SseB-Chi-C48/80+BECC438b had a significant increase in MFI for P2X7 (**Figure 5**). Therefore, in conjunction with the findings above, we determined that the optimal dose was on the order of 20 µg L-SseB formulated with Chi-C48/80+BECC438b.

### Determining the vaccine-mediated reduction in bacterial loads in different organs

**Figure 6:**
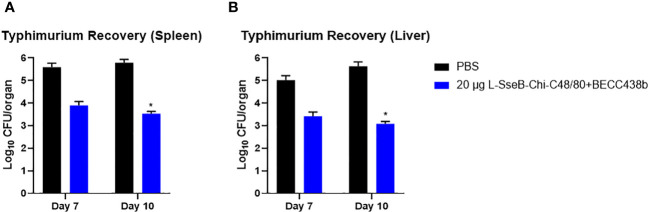
Determination of bacterial organ load in immunized mice. Balb/C mice (n=5/group) were immunized with PBS or L-SseB with the stated formulation on days 0, 14 and 28. On day 56, the mice were challenged orally with *S.* Typhimurium (1 x 10^6^ CFU/mouse). Mice were euthanized on days 7 and 10 post infection and the bacterial loads in the spleen **(A)** and liver **(B)** were determined. Significance was calculated by comparing CFU from PBS vaccinated mice to CFU from the L-SseB vaccinated groups by using one-way ANOVA (Dunnett’s multiple comparison test) for respective time points and organs. *p<0.05.

To test the efficacy of 20 µg L-SseB-Chi-C48/80+BECC438b, its ability to reduce bacterial loads within the target organs was determined. Two sets of mice were vaccinated with 20 µg L-SseB-Chi-C48/80+BECC438b or PBS and challenged with *S.* Typhimurium at a sub-lethal dose (3x10^5^ CFU). Livers and spleens were harvested on days 7 and 10, single cell suspensions were prepared and dilution plating with streptomycin selection was performed. On day 7, a reduced bacterial load was observed in both the liver and spleen of mice vaccinated with 20 µg L-SseB-Chi-C48/80+BECC438b when compared to PBS vaccinated mice; however, while it was a two-log reduction in bacteria it was not statistically significant. On day 10, a further reduction in bacterial load was observed in 20 µg L-SseB-Chi-C48/80+BECC438b vaccinated mice in both liver and spleen and this was determined to be significant relative to the PBS vaccinated mice (**Figure 6**). These findings shown that vaccination with 20 µg L-SseB-Chi-C48/80+BECC438b reduces disease severity by reducing the bacterial load in the visceral organs. In addition to the survival study (**Figure 1**), this challenge experiment shows that the 20 µg L-SseB-Chi-C48/80+BECC438b is protective by efficiently reducing the bacterial loads in the affected organs.

### Cytokine production in response to stimulation with SseB in immunized mice

**Figure 7:**
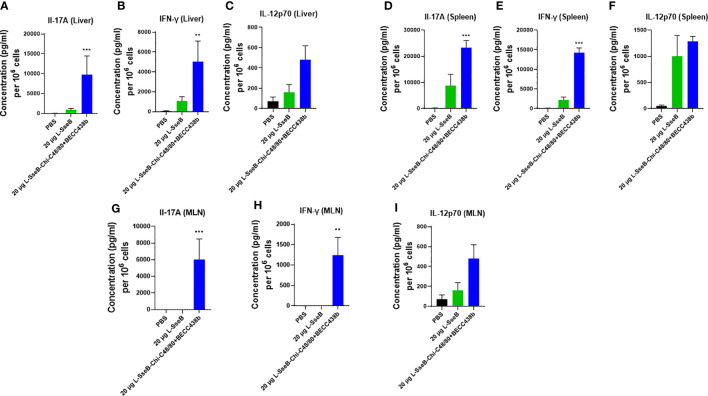
Cytokine secretion by isolated cells in response to stimulation with SseB. Balb/C mice (n=4/group) were immunized IN with PBS, 20 µg L-SseB or 20 µg L-SseB-Chi-C48/80+BECC438b on days 0, 14 and 28. On day 56, the mice were euthanized, and the liver, spleen and mesenteric lymph nodes (MLN) processed to obtain single cell suspensions. Single cell suspensions of livers **(A–C)**, spleens **(D–F)** and MLNs **(G–I)** were stimulated with SseB. The supernatants were analyzed for cytokine production by MSD analysis as per manufacturer’s specification. Significance was calculated by comparing cytokine secretion of cells isolated from PBS immunized mice to all vaccinated groups by using one-way ANOVA (Dunnett’s multiple comparison test). **p<0.01, ***p<0.001.

To better understand the mechanism by which the Chi-C48/80+BECC438b heightens the immunogenicity and protective efficacy of L-SseB, groups of mice were immunized with PBS, 20 µg L-SseB alone, and 20ug L-SseB with Chi-C48/80+BECC438b. On day 56 post immunization, mice were sacrificed, single cell suspensions of liver, spleen, and mesenteric lymph nodes (MLN) were prepared, and the cells were stimulated with SseB. After 48 h, the supernatants were collected and analyzed for secreted cytokines. It was observed that L-SseB-Chi-C48/80+BECC438b induced significantly higher level of secreted IL-17A and IFN-γ than did L-SseB alone in all three organs (**Figure 7**). IL-12p70 secretion from the L-SseB-Chi-C48/80+BECC438b was again elevated in all three organs but these differences were found not to be significant.

### Validation of vaccine efficacy in a mouse *S*. Typhi infection model

**Figure 8:**
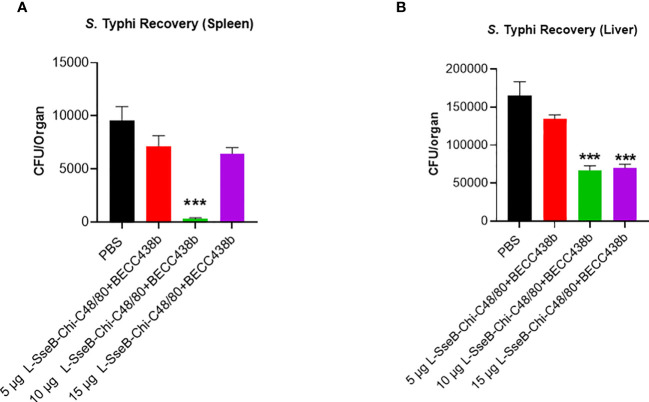
Validation of vaccine efficacy in a mouse Typhoid model. Balb/C mice (n=5/group) were immunized with PBS or 5, 10, 15 µg L-SseB-Chi-C48/80-BECC438b on days 0, 14 and 28. On day 56, mice were challenged orally with *S.* Typhi (1 x 10^4^ CFU/mouse). The mice were euthanized on day 3 post infection and the bacterial load in spleen **(A)** and liver **(B)** was evaluated. Significance was calculated by comparing CFU from PBS immunized mice to CFU from vaccinated groups by using one-way ANOVA (Dunnett’s multiple comparison test) for respective time points and organs. ***p<0.001.

Using the iron-overload mouse model (27) the efficacy of the 5, 10, and 15 µg L-SseB-Chi-C48/80+BECC438b was tested against *S.* Typhi (the causative agent of the systemic febrile illness in humans). Compared to the PBS immunized group, a statistically significant reduction in bacterial load in spleen was observed with 10 µg L-SseB-Chi-C48/80+BECC438b, with visible reduction in the other groups (**Figure 8**). Bacterial loads in the liver were significantly reduced for both the 10 µg and 15 µg L-SseB with Chi-C48/80+BECC438b formulations. These observations point to the ability of the vaccine to reduce bacterial burden and therefore disease severity in these mice. This confirms that vaccines developed using the L-SseB platform should be broadly protective across *Salmonella* serovars.

### Evaluation of the L-SseB-Chi-C48/80+BECC438b formulation in a rabbit infection model

**Figure 9:**
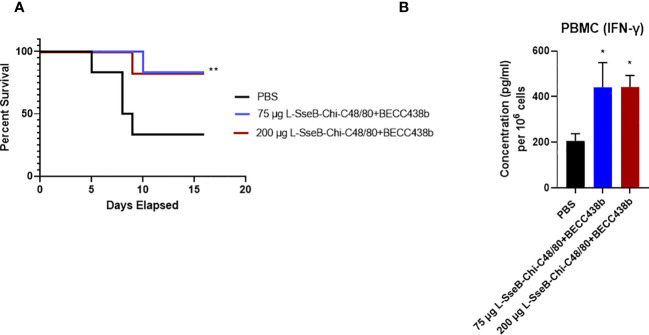
Evaluation of the protective efficacy for the chitosan-based NP formulation in rabbits. Rabbits (n=6/group) were immunized IN with PBS or Chi-C48/80-BECC438b with either 75 µg or 200 µg L-SseB on days 0, 14 and 28. On day 56 they were orally challenged with 1x 10^6^ CFU of *S.* Typhimurium and observed for moribundity and euthanized according to pre-determined and IACUC-approved endpoints. **(A)** Survival was plotted on a Kaplan-Meier graph. Significance was calculated by comparing survival of individual vaccinated groups with the PBS vaccinated group using Log-rank (Mantel-Cox) test. *p<0.05, **p<0.01. **(B)** Rabbits were bled on day 42 post vaccination and PBMCs were isolated. The isolated PBMCs were stimulated with SseB and cultured for 24 hours. IFN-γ secretion into the supernatant was determined by ELISA. Significance was calculated by comparing cytokine secretion by cells isolated from PBS vaccinated mice to the two vaccinated groups using a one-way ANOVA (Dunnett’s multiple comparison test). *p<0.05, **p<0.01.

Having tested the immunogenicity and efficacy in rodents, the findings were tested in a rabbit infection model for validation. Groups of rabbits were immunized IN with PBS, 75 µg L-SseB-Chi-C48/80+BECC438b or 200 µg L-SseB-Chi-C48/80+BECC438b on days 0, 14, and 28. On day 56, they were challenged orally with *S.* Typhimurium and monitored for diarrhea and morbidity. Rabbits vaccinated with 75 µg or 200 µg L-SseB admixed with Chi-C48/80+BECC438b were protected against *S.* Typhimurium infection when compared to PBS vaccinated rabbits (**Figure 9A**). In addition, rabbits vaccinated with 75 µg L-SseB-Chi-C48/80+BECC438b lost less weight and recovered faster than both the PBS and 200 µg L-SseB-Chi-C48/80+BECC438b immunized rabbits (data not shown). The data show that L-SseB-Chi-C48/80+BECC438b is able to provide significant protection in the rabbit model of infection.

To evaluate the immune responses for the rabbits upon vaccination, they were bled on day 42 and PBMCs were isolated and incubated with SseB. After 24 h, the supernatants were analyzed for cytokine secretion. Significant IFN-γ secretion was observed in PBMC’s isolated from rabbits immunized with both doses of L-SseB-Chi-C48/80+BECC438b (**Figure 9B**). Interestingly, in this case, there was no significant secretion of IL-17A seen for the PCMBs from any of the groups. As discussed earlier, IFN-γ is important for the control of infection in later stages. Although no IL-17A was detected, this may be because this experiment was performed using isolated PBMCs whereas all previous studies were performed in reticuloendothelial organs (liver and spleen) and MLNs.

## Discussion

SseB has been reported to protect mice against a *S.* Typhimurium challenge when delivered via the intravenous route in the presence of LPS and or flagellin (18, 31). In the work presented here, we demonstrate that SseB can be delivered intranasally fused to LTA1, a potent mucosal adjuvant to elicit protection. In this case, protection was optimal when the L-SseB was formulated with a polysaccharide nanoparticle (Chi-C48/80) and a TLR4 agonist (BECC438b). The advantage of presenting L-SseB on a nanoparticle is that it allows for multimeric presentation of antigen, and this may be important when adapting this vaccine candidate for use in humans. Furthermore, the inclusion of BECC438b provides a proven TLR4 agonist that is a biosimilar of MPL which has been approved for use in some human vaccine formulations (e.g. vaccines against shingles and human papillomavirus). Furthermore, the presence of the LTA1 moiety and BECC438b might be expected to work cooperatively to elicit significant IL-17 and IFN-γ responses in the liver, spleen, and MLN as well as the presence of TRM in the liver, aspects of this formulation that were tested here.

The subclass of T cells secreting IL-17 (Th17 cells) have been reported to play an important role in imparting immunity to extracellular bacteria (36, 37) The importance of IL-17 is further supported by the fact that mice deficient in IL-17 have increased bacterial burden in visceral organs compared to wildtype mice (38). Furthermore, IL-17 deficient mice are also unable to recruit neutrophils to the intestine during infection which may contribute to higher bacterial burden (39). In summary, IL-17 plays an important role in the protection of the intestinal mucosa. Cells isolated from mice vaccinated with L-SseB-Chi-C48/80-BECC438b did demonstrate significantly higher IL-17A levels following SseB stimulation which agrees with these previous observations.

Studies have shown the correlation of *Salmonella-*specific CD4 and CD8 T cells with a positive outcome of vaccination with the licensed typhoidal vaccines (40, 41) *Salmonella* specific CD4 T cells can secrete IFN-γ in response to infection and are capable of homing to sites of infection (42, 43). Moreover, studies have provided insights about the age-dependency of vaccine efficacy in the context of tissue resident memory T cell responsiveness (44). IFN-γ secreted at the sites of infection then activates macrophages to aid in control of infection. IL-12 is a key IFN-γ inducing cytokine which has a major role in immunity against intracellular pathogens (45). Once again, these prior observations agree with the cytokine prolife induced by L-SseB-Chi-C48/80-BECC348b as being a major factor in protecting vaccinated from lethal *Salmonella* infection. In the same respect, CD4^+^CD69^+^ T cells expressing a high level of P2X7 have previously been characterized as TRMs that are key factors in promoting protection against the severe effects of *Salmonella* infection (28). This subset of non-circulating T cells reside in the liver of protected mice and others have shown that unvaccinated mice are partially protected upon adaptive transfer of these cell (28). Taken together, the combined data presented here show that L-SseB-Chi-C48/80-BECC438b induces a significant systemic (liver and spleen) as well as gut-directed (MLN) immune response. Although, the expression of the gut homing receptor was not studied, the fact that there are increased numbers of SseB-specific cytokine secreting cells in the MLN of the mice immunized with L-SseB-Chi-C48/80-BECC438b suggests that homing is occurring.

The observations presented here strongly suggest that a proposed chitosan-based nanoparticle vaccine has the potential to be a “pan-*Salmonella*” vaccine, effective against both typhoidal and non-typhoidal *Salmonella* (NTS) serovars. This is especially important because there are no licensed vaccines available for invasive NTS, which has been identified as the leading cause of bacteremia in sub-Saharan Africa (3, 6, 7). High risk groups include children with malaria and malnutrition or HIV infection along with immunocompromised adults (3, 6, 7). Clinically iNTS presents as a febrile illness without diarrhea and leads to invasive bacteremia not limited to the intestine (3, 6, 7). This coupled with the emerging multi-drug resistant strains highlight the need for a vaccine effective across serovars (3, 6, 7) such as the one described in this work.

Herein, we outline the process of selecting an efficacious formulation, choosing the optimum dose for the antigen, immunological studies, and finally validation in a non-rodent model. It should also be noted that the vaccine was evaluated to be efficacious against both a typhoidal and non-typhoidal *Salmonella* serovar. Although L-SseB alone and all the more complex formulations were able to generate comparable high antibody titers against SseB (**Supplementary Figures 2-4**), this was not sufficient to ensure protection. As reported by others, we found that TRMs in conjunction with the activation of IL-17A, IFN-γ and IL-12p70 secreting cells are collectively needed for protection against *Salmonella* infection in mice. To build upon these important findings, however, the contribution of each of the players in protection needs to be studied in greater detail. This work is an early steppingstone for designing future studies that will facilitate the prediction of corelates of protection for a successful vaccine.

## Data availability statement

The original contributions presented in the study are included in the article/**Supplementary Material**. Further inquiries can be directed to the corresponding author.

## Ethics statement

The animal study was reviewed and approved by University of Kansas Institutional Animal Care and Use Committee Practices.

## Author contributions

SD designed and completed most of the animal experiments and the immunological studies, was closely involved with experimental design and performed the data analysis. He also wrote the first draft of this paper. DH was also a major contributor in completing the animal experiments, designing the animal studies, collecting samples and data analysis/interpretation. He also contributed to the editing of the initial drafts of this paper. TL assisted with the animal experiments and contributed to interpreting and analyzing the data. SW and SR were responsible for the cloning expression, purification and biochemical analysis of SseB and L-SseB. GH AND SS were responsible for the biophysical analysis of SseB and L-SseB and the preparation of the NP formulations used for immunization. DV and RE prepared and provided the BECC438b. They were also involved in discussions of the data. ZD was instrumental in sample preparation and the handling of the mice. WDP was involved in the interpretation of the data and editing/finalizing the manuscript. WLP conceived the overall investigation and was instrumental in designing the overall study and providing funding for this work. All authors contributed to the article and approved the submitted version.

## References

[1] Batz MB Hoffmann S Morris JG Jr. Ranking the disease burden of 14 pathogens in food sources in the united states using attribution data from outbreak investigations and expert elicitation J Food Prot (2012) 75(7):1278–91 doi: 10.4315/0362-028X.JFP-11-418 22980012

[2] Andrews-Polymenis HL Baumler AJ McCormick BA Fang FC. Taming the elephant: salmonella biology, pathogenesis, and prevention. Infect Immun (2010) 78(6):2356–69. doi: 10.1128/IAI.00096-10 PMC2876576 20385760

[3] Collaborators GBDN-TSID. The global burden of non-typhoidal salmonella invasive disease: a systematic analysis for the global burden of disease study 2017. Lancet Infect Dis (2019) 19(12):1312–24. doi: 10.1016/S1473-3099(19)30418-9 PMC6892270 31562022

[4] Medalla F Gu W Friedman CR Judd M Folster J Griffin PM. Increased incidence of antimicrobial-resistant nontyphoidal salmonella infections, united states, 2004-2016. Emerg Infect Dis (2021) 27(6):1662–72. doi: 10.3201/eid2706.204486 PMC8153855 34013877

[5] Chirwa EB Dale H Gordon MA Ashton PM. What is the source of infections causing invasive nontyphoidal salmonella disease? Open Forum Infect Dis (2023) 10(3):ofad086. doi: 10.1093/ofid/ofad086 36910696 PMC10004642

[6] Uche IV MacLennan CA Saul A. A systematic review of the incidence, risk factors and case fatality rates of invasive nontyphoidal salmonella (Ints) disease in Africa (1966 to 2014). PloS Negl Trop Dis (2017) 11(1):e0005118. doi: 10.1371/journal.pntd.0005118 28056035 PMC5215826

[7] Crump JA Heyderman RS. A perspective on invasive salmonella disease in Africa. Clin Infect Dis (2015) 61 Suppl 4:S235–40. doi: 10.1093/cid/civ709 PMC4596931 26449937

[8] Koolman L Prakash R Diness Y Msefula C Nyirenda TS Olgemoeller F. Case-control investigation of invasive salmonella disease in Malawi reveals no evidence of environmental or animal transmission of invasive strains, and supports human to human transmission. PloS Negl Trop Dis (2022) 16(12):e0010982. doi: 10.1371/journal.pntd.0010982 36508466 PMC9779717

[9] Kingsley RA Msefula CL Thomson NR Kariuki S Holt KE Gordon MA. Epidemic multiple drug resistant salmonella typhimurium causing invasive disease in Sub-Saharan Africa have a distinct genotype. Genome Res (2009) 19(12):2279–87. doi: 10.1101/gr.091017.109 PMC2792184 19901036

[10] Galen JE Pasetti MF Tennant S Ruiz-Olvera P Sztein MB Levine MM. Salmonella enterica serovar typhi live vector vaccines finally come of age. Immunol Cell Biol (2009) 87(5):400–12. doi: 10.1038/icb.2009.31 PMC3747779 19417771

[11] Carlin NI Svenson SB Lindberg AA. Role of monoclonal O-antigen antibody epitope specificity and isotype in protection against experimental mouse typhoid. Microb Pathog (1987) 2(3):171–83. doi: 10.1016/0882-4010(87)90019-2 2467161

[12] Svenson SB Lindberg AA. Artificial salmonella vaccines: salmonella typhimurium O-Antigen-Specific oligosaccharide-protein conjugates elicit protective antibodies in rabbits and mice. Infect Immun (1981) 32(2):490–6. doi: 10.1128/iai.32.2.490-496.1981 PMC351472 6166555

[13] Simon R Tennant SM Wang JY Schmidlein PJ Lees A Ernst RK. Salmonella enterica serovar enteritidis core O polysaccharide conjugated to H:G,M flagellin as a candidate vaccine for protection against invasive infection with s. Enteritidis. Infect Immun (2011) 79(10):4240–9. doi: 10.1128/IAI.05484-11 PMC3187246 21807909

[14] McSorley SJ. Immunity to intestinal pathogens: lessons learned from salmonella. Immunol Rev (2014) 260(1):168–82. doi: 10.1111/imr.12184 PMC4066191 24942689

[15] Darton TC Jones C Blohmke CJ Waddington CS Zhou L Peters A. Using a human challenge model of infection to measure vaccine efficacy: a randomised, controlled trial comparing the typhoid vaccines M01zh09 with placebo and Ty21a. PloS Negl Trop Dis (2016) 10(8):e0004926. doi: 10.1371/journal.pntd.0004926 27533046 PMC4988630

[16] Jin C Gibani MM Moore M Juel HB Jones E Meiring J. Efficacy and immunogenicity of a vi-tetanus toxoid conjugate vaccine in the prevention of typhoid fever using a controlled human infection model of salmonella typhi: a randomised controlled, phase 2b trial. Lancet (2017) 390(10111):2472–80. doi: 10.1016/S0140-6736(17)32149-9 PMC5720597 28965718

[17] Juel HB Thomaides-Brears HB Darton TC Jones C Jones E Shrestha S. Salmonella typhi bactericidal antibodies reduce disease severity but do not protect against typhoid fever in a controlled human infection model. Front Immunol (2017) 8:1916. doi: 10.3389/fimmu.2017.01916 29387052 PMC5776093

[18] Lee SJ Liang L Juarez S Nanton MR Gondwe EN Msefula CL. Identification of a common immune signature in murine and human systemic salmonellosis. Proc Natl Acad Sci U.S.A. (2012) 109(13):4998–5003. doi: 10.1073/pnas.1111413109 22331879 PMC3324033

[19] Clements JD Norton EB. The mucosal vaccine adjuvant Lt(R192g/L211a) or dmlt. mSphere (2018) 3(4). doi: 10.1128/mSphere.00215-18 PMC6060342 30045966

[20] Gregg KA Harberts E Gardner FM Pelletier MR Cayatte C Yu L. A lipid a-based Tlr4 mimetic effectively adjuvants a *Yersinia pestis* Rf-V1 subunit vaccine in a murine challenge model. Vaccine (2018) 36(28):4023–31. doi: 10.1016/j.vaccine.2018.05.101 PMC6057149 29861179

[21] Das S Howlader DR Zheng Q Ratnakaram SSK Whittier SK Lu T. Development of a broadly protective, self-adjuvanting subunit vaccine to prevent infections by *Pseudomonas aeruginosa* . Front Immunol (2020) 11:583008. doi: 10.3389/fimmu.2020.583008 33281815 PMC7705240

[22] Anderson M Sansonetti PJ Marteyn BS. Shigella diversity and changing landscape: insights for the twenty-first century. Front Cell Infect Microbiol (2016) 6:45. doi: 10.3389/fcimb.2016.00045 27148494 PMC4835486

[23] Martinez-Becerra FJ Kissmann JM Diaz-McNair J Choudhari SP Quick AM Mellado-Sanchez G. Broadly protective *Shigella* vaccine based on type iii secretion apparatus proteins. Infect Immun (2012) 80(3):1222–31. doi: 10.1128/IAI.06174-11 PMC3294653 22202122

[24] Howlader DR Das S Lu T Hu G Varisco DJ Dietz ZK. Effect of two unique nanoparticle formulations on the efficacy of a broadly protective vaccine against pseudomonas aeruginosa. Front Pharmacol (2021) 12:706157. doi: 10.3389/fphar.2021.706157 34483911 PMC8416447

[25] Iyer V Cayatte C Guzman B Schneider-Ohrum K Matuszak R Snell A. Impact of formulation and particle size on stability and immunogenicity of oil-in-Water emulsion adjuvants. Hum Vaccines immunotherapeutics (2015) 11(7):1853–64. doi: 10.1080/21645515.2015.1046660 PMC4517459 26090563

[26] Robertsson JA Lindberg AA Hoiseth S Stocker BA. Salmonella typhimurium infection in calves: protection and survival of virulent challenge bacteria after immunization with live or inactivated vaccines. Infect Immun (1983) 41(2):742–50. doi: 10.1128/iai.41.2.742-750.1983 PMC264704 6347895

[27] Das S Chowdhury R Pal A Okamoto K Das S. Salmonella typhi outer membrane protein stiv is a potential candidate for vaccine development against typhoid and paratyphoid fever. Immunobiology (2019) 224(3):371–82. doi: 10.1016/j.imbio.2019.02.011 30952553

[28] Benoun JM Peres NG Wang N Pham OH Rudisill VL Fogassy ZN. Optimal protection against salmonella infection requires noncirculating memory. Proc Natl Acad Sci U.S.A. (2018) 115(41):10416–21. doi: 10.1073/pnas.1808339115 PMC6187142 30254173

[29] Hanes DE Robl MG Schneider CM Burr DH. New Zealand white rabbit as a nonsurgical experimental model for salmonella enterica gastroenteritis. Infect Immun (2001) 69(10):6523–6. doi: 10.1128/IAI.69.10.6523-6526.2001 PMC98790 11553599

[30] Martinez-Becerra FJ Kumar P Vishwakarma V Kim JH Arizmendi O Middaugh CR. Characterization and protective efficacy of type iii secretion proteins as a broadly protective subunit vaccine against salmonella enterica serotypes. Infect Immun (2018) 86(3). doi: 10.1128/IAI.00473-17 PMC5820934 29311233

[31] Lee SJ Benoun J Sheridan BS Fogassy Z Pham O Pham QM. Dual immunization with Sseb/Flagellin provides enhanced protection against salmonella infection mediated by circulating memory cells. J Immunol (2017) 199(4):1353–61. doi: 10.4049/jimmunol.1601357 PMC5548602 28710253

[32] Lu T Das S Howlader DR Zheng Q Siva Sai Kumar R Whittier SK. L-dbf elicits cross protection against different serotypes of *Shigella* spp. Front Trop Dis (2021) 2. doi: 10.3389/fitd.2021.729731

[33] Corthesy B Bioley G. Lipid-based particles: versatile delivery systems for mucosal vaccination against infection. Front Immunol (2018) 9:431. doi: 10.3389/fimmu.2018.00431 29563912 PMC5845866

[34] Marasini N Skwarczynski M Toth I. Intranasal delivery of nanoparticle-based vaccines. Ther Delivery (2017) 8(3):151–67. doi: 10.4155/tde-2016-0068 28145824

[35] Holzer SU Hensel M. Functional dissection of translocon proteins of the salmonella pathogenicity island 2-encoded type iii secretion system. BMC Microbiol (2010) 10:104. doi: 10.1186/1471-2180-10-104 20377892 PMC2873485

[36] Weaver CT Hatton RD Mangan PR Harrington LE. Il-17 family cytokines and the expanding diversity of effector T cell lineages. Annu Rev Immunol (2007) 25:821–52. doi: 10.1146/annurev.immunol.25.022106.141557 17201677

[37] Curtis MM Way SS. Interleukin-17 in host defence against bacterial, mycobacterial and fungal pathogens. Immunology (2009) 126(2):177–85. doi: 10.1111/j.1365-2567.2008.03017.x PMC2632692 19125888

[38] Schulz SM Kohler G Schutze N Knauer J Straubinger RK Chackerian AA. Protective immunity to systemic infection with attenuated salmonella enterica serovar enteritidis in the absence of il-12 is associated with il-23-Dependent il-22, but not il-17. J Immunol (2008) 181(11):7891–901. doi: 10.4049/jimmunol.181.11.7891 19017979

[39] Raffatellu M Santos RL Verhoeven DE George MD Wilson RP Winter SE. Simian immunodeficiency virus-induced mucosal interleukin-17 deficiency promotes salmonella dissemination from the gut. Nat Med (2008) 14(4):421–8. doi: 10.1038/nm1743 PMC2901863 18376406

[40] Booth JS Goldberg E Barnes RS Greenwald BD Sztein MB. Oral typhoid vaccine Ty21a elicits antigen-specific resident memory Cd4(+) T cells in the human terminal ileum lamina propria and epithelial compartments. J Transl Med (2020) 18(1):102. doi: 10.1186/s12967-020-02263-6 32098623 PMC7043047

[41] Booth JS Patil SA Goldberg E Barnes RS Greenwald BD Sztein MB. Attenuated oral typhoid vaccine Ty21a elicits lamina propria and intra-epithelial lymphocyte tissue-resident effector memory Cd8 T responses in the human terminal ileum. Front Immunol (2019) 10:424. doi: 10.3389/fimmu.2019.00424 30923521 PMC6426796

[42] Mittrucker HW Kohler A Kaufmann SH. Characterization of the murine T-lymphocyte response to salmonella enterica serovar typhimurium infection. Infect Immun (2002) 70(1):199–203. doi: 10.1128/IAI.70.1.199-203.2002 11748183 PMC127597

[43] Srinivasan A Foley J McSorley SJ. Massive number of antigen-specific Cd4 T cells during vaccination with live attenuated salmonella causes interclonal competition. J Immunol (2004) 172(11):6884–93. doi: 10.4049/jimmunol.172.11.6884 15153507

[44] Booth JS Goldberg E Patil SA Barnes RS Greenwald BD Sztein MB. Age-dependency of terminal ileum tissue resident memory T cell responsiveness profiles to s. typhi following oral Ty21a immunization in humans. Immun Ageing (2021) 18(1):19. doi: 10.1186/s12979-021-00227-y 33874975 PMC8053564

[45] Fieschi C Casanova JL. The role of interleukin-12 in human infectious diseases: only a faint signature. Eur J Immunol (2003) 33(6):1461–4. doi: 10.1002/eji.200324038 12778462

