# Supplementary material for: Immunogenicity and protective efficacy of nanoparticle formulations of L-SseB against Salmonella infection
Source: Front Immunol. 2023 Jun 30;14:1208848. doi: 10.3389/fimmu.2023.1208848 (PMC10347375; doi:10.3389/fimmu.2023.1208848)
Supplement: Supplementary file 1 [file DataSheet_1.pdf]

Supplemental Materials for:

Immunogenicity and protective efficacy of nanoparticle formulations of L-SseB against *Salmonella* infection

Sayan Das[1†], Debaki R. Howlader[1‡], Ti Lu[1‡], Sean K. Whittier[1‡], Gang Hu[1], Siva S. K. Ratnakaram[1π], David J Varisco[2], Zackary K. Dietz[1‡], Robert K. Ernst[2], William D. Picking[1‡], and Wendy L. Picking[1‡*]

[1]*Department of Pharmaceutical Chemistry, University of Kansas, Lawrence, KS 66047, and*

[2]*Department of Microbial Pathogenesis, University of Maryland, Baltimore, MD 21201*

[*]Corresponding author:

Wendy Picking, wendy.picking@missouri.edu

Current address: [†]Department of Microbial Pathogenesis, University of Maryland, Baltimore, MD 21201; [‡]Department of Veterinary Pathobiology and Bond Life Science Center, University of Missouri, Columbia, MO 65211; [π]Integrated DNA Technologies, Coralville Iowa

Keywords: *Salmonella,* T3SS, Vaccine, Formulation, Typhoid, IL-17

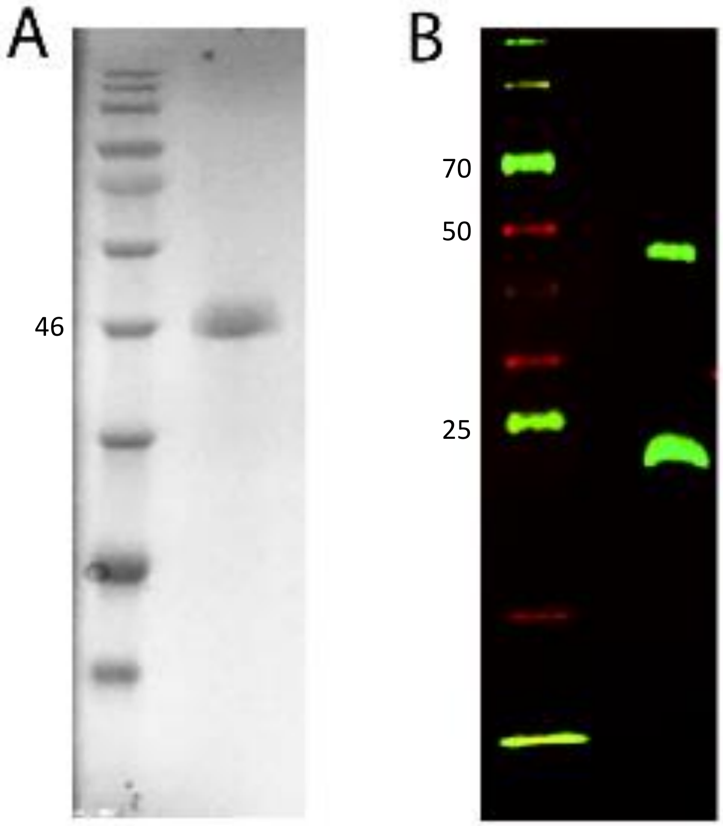

Supplemental Figure S1. L-SseB and ADP-ribosylation activity. A) L-SseB was purified as described and is shown after separation using SDS-PAGE (0.05 µg) and staining with One-Step Blue protein gel stain (Biotium). B) The LTA1 moiety of L-SseB was then shown to transfer biotin-ADPr from biotin-NAD$^+$ to itself and ARF4. The biotin-ADPr staining was visualized using western blot analysis followed by probing with Streptavidin–IR800.

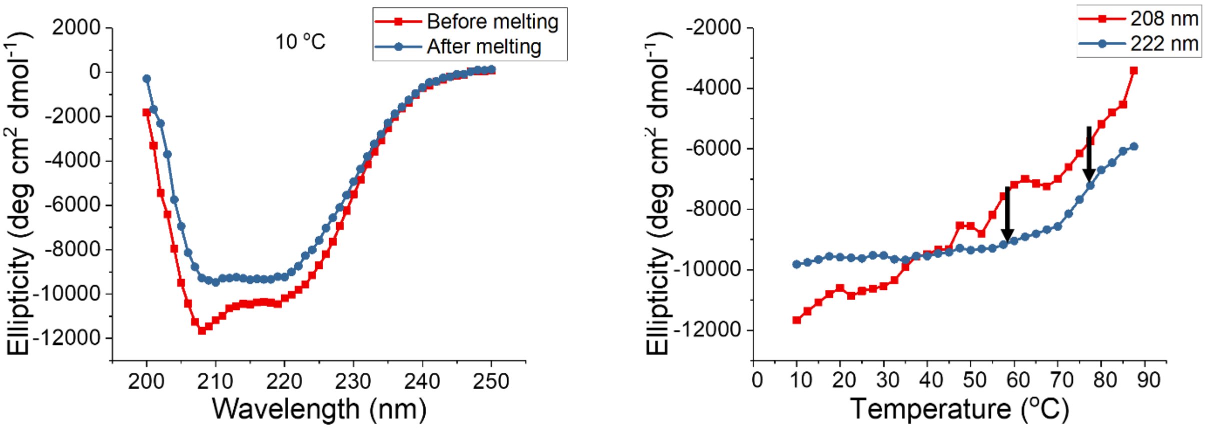

Supplemental Figure S2. Refolded L-SseB has an ordered secondary structure. The circular dichroism (CD) spectrum of L-SseB indicates a mixed secondary structure when refolded after IMAC purification in the presence of high concentrations of urea (left panel, blue line). It contains an estimated 25% α-helices, 27% β-strands, 12% turns and 36% other (random, etc.). When subjected to thermal unfolding and the signals at 208 and 222 nm are monitored, there is a temperature-dependent loss of α-helical structure and a possible gain of β-structure, which suggests that aggregation may occur at high temperatures (right panel). When cooled back to 10 ˚C, the CD spectrum suggest some refolding to the native state by some loss of the original structure, possibly due to aggregation. Nevertheless, the major transitions seen 60 ˚C (208 nm) and 70 to 75 ˚C show that the purified L-SseB is highly stable following IMAC purification and renaturation.

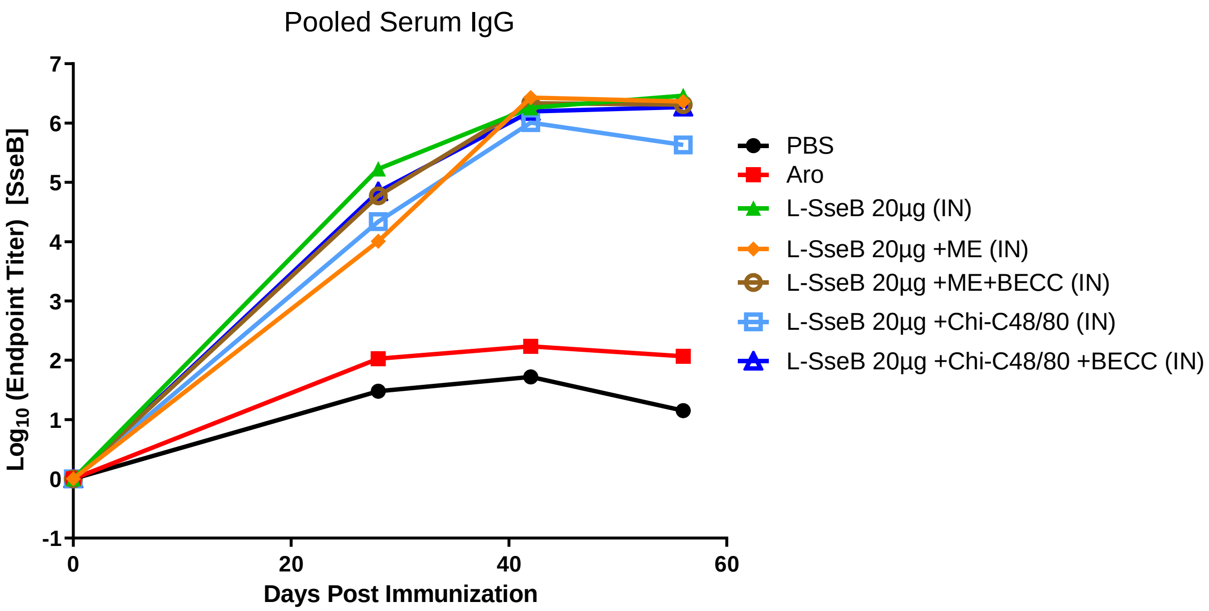

Supplemental Figure S3. Kinetics of serum IgG response to formulations of L-SseB. Mice were vaccinated IN three times (days 0, 14, and 28). Blood samples were collected at indicated time points and pooled serum titers specific for SseB were measured by ELISA.

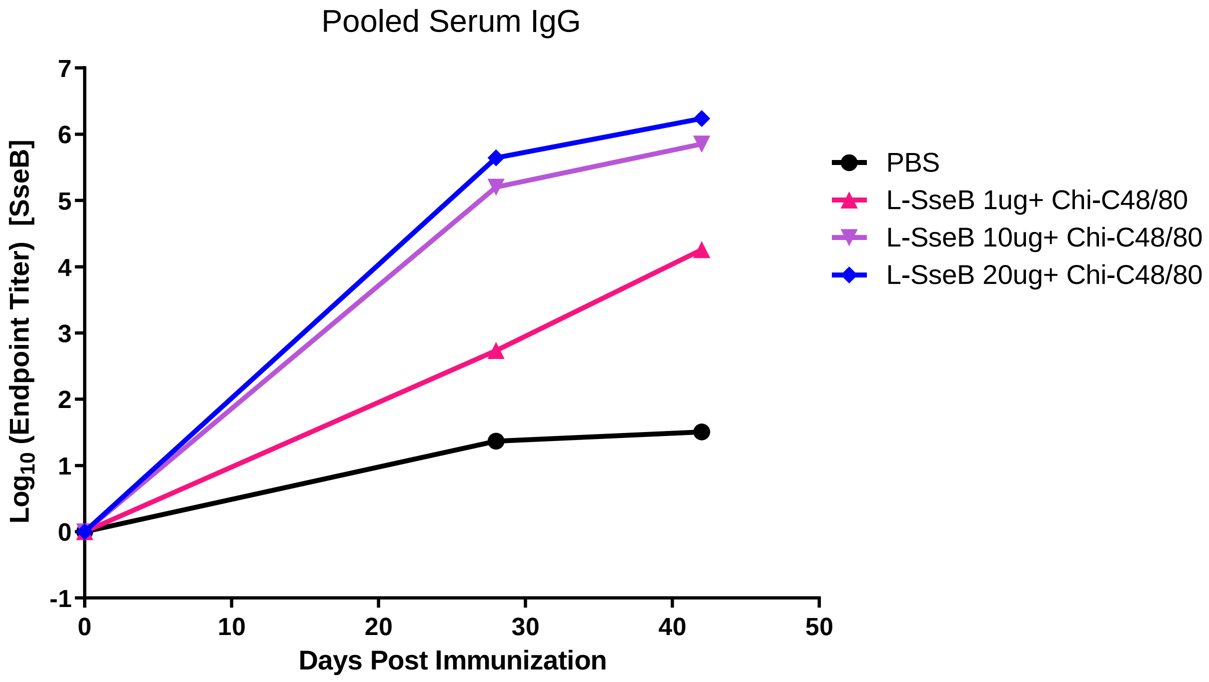

Supplemental Figure S4. Kinetics of serum IgG response to dose escalation. Mice were vaccinated IN three times (days 0, 14, and 28). Blood samples were collected at indicated time points and pooled serum titers specific for SseB were measured by ELISA.

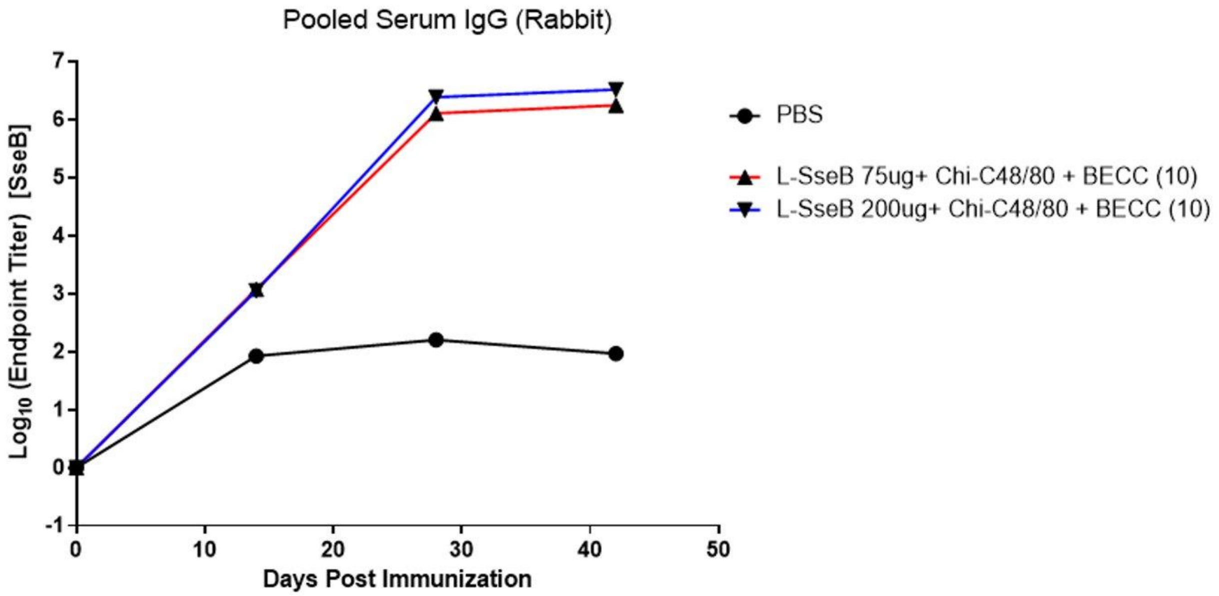

Supplemental Figure S5. Kinetics of serum IgG response. Rabbits were vaccinated IN three times (days 0, 14, and 28). Blood samples were collected at indicated time points and pooled serum titers specific for SseB were measured by ELISA.
